# ClotCatcher: a novel natural language model to accurately adjudicate venous thromboembolism from radiology reports

**DOI:** 10.1186/s12911-023-02369-z

**Published:** 2023-11-16

**Authors:** Jeffrey Wang, Joao Souza de Vale, Saransh Gupta, Pulakesh Upadhyaya, Felipe A. Lisboa, Seth A. Schobel, Eric A. Elster, Christopher J. Dente, Timothy G. Buchman, Rishikesan Kamaleswaran

**Affiliations:** 1 Department of Biomedical Informatics, Emory University School of Medicine, 1462 Clifton Road, Suite 504, Atlanta, GA 30322 USA; 2 Surgical Critical Care Initiative (SC2i), Uniformed Services University of the Health Sciences, Bethesda, MD 20814 USA; 3 Department of Surgery, Uniformed Services University of the Health Sciences and Walter Reed National Military Medical Center, Bethesda, MD 20814 USA; 4 Henry M. Jackson Foundation for the Advancement of Military Medicine, Inc., Bethesda, MD 20817 USA; 5 Emory Department of Surgery, Emory University School of Medicine, Atlanta, GA USA; 6 Grady Memorial Hospital, Atlanta, GA USA; 7 Emory Critical Care Center, Atlanta, GA USA

**Keywords:** Natural language processing, Machine learning, Venous thromboembolism

## Abstract

**Introduction:**

Accurate identification of venous thromboembolism (VTE) is critical to develop replicable epidemiological studies and rigorous predictions models. Traditionally, VTE studies have relied on international classification of diseases (ICD) codes which are inaccurate – leading to misclassification bias. Here, we developed ClotCatcher, a novel deep learning model that uses natural language processing to detect VTE from radiology reports.

**Methods:**

Radiology reports to detect VTE were obtained from patients admitted to Emory University Hospital (EUH) and Grady Memorial Hospital (GMH). Data augmentation was performed using the Google PEGASUS paraphraser. This data was then used to fine-tune ClotCatcher, a novel deep learning model. ClotCatcher was validated on both the EUH dataset alone and GMH dataset alone.

**Results:**

The dataset contained 1358 studies from EUH and 915 studies from GMH (*n* = 2273). The dataset contained 1506 ultrasound studies with 528 (35.1%) studies positive for VTE, and 767 CT studies with 91 (11.9%) positive for VTE. When validated on the EUH dataset, ClotCatcher performed best (AUC = 0.980) when trained on both EUH and GMH dataset without paraphrasing. When validated on the GMH dataset, ClotCatcher performed best (AUC = 0.995) when trained on both EUH and GMH dataset with paraphrasing.

**Conclusion:**

ClotCatcher, a novel deep learning model with data augmentation rapidly and accurately adjudicated the presence of VTE from radiology reports. Applying ClotCatcher to large databases would allow for rapid and accurate adjudication of incident VTE. This would reduce misclassification bias and form the foundation for future studies to estimate individual risk for patient to develop incident VTE.

**Supplementary Information:**

The online version contains supplementary material available at 10.1186/s12911-023-02369-z.

## Introduction

Venous thromboembolism (VTE) is defined as the development of either a deep venous thrombus (DVT) or pulmonary embolism (PE) and is widely considered to be a preventable and leading cause of death worldwide [1–3]. VTE is estimated to occur in 1 in 1000 patients in the United States [4] and is associated with increased cost [5], hospital length of stay [6], morbidity [7], and a higher risk of both short-term and long-term mortality [8]. There have been several nation-wide campaigns addressing VTE as a public health issue, including a call in 2008 to prevent in-hospital VTE by the United States Surgeon General [9, 10].

Several studies utilizing large electronic medical record databases to determine predictors and outcomes in patients who develop VTE have recently been published; however, they are limited by their reliance on using International Classification of Disease (ICD) codes to determine the incidence of VTE [11–13]. While ICD codes are easily accessible and can be rapidly applied to large databases, they have several important limitations. First, their accuracy has been called into question with recent analyses finding that among patients with an ICD diagnosis of VTE, only 30%—60% have clinical documentation from radiological reports to support this diagnosis [12, 14, 15]. Another important limitation is that ICD codes do not provide information regarding the time of incident VTE, as it can only be used to identify whether the condition was present or absent. Without data on timing, analysis and modeling are limited to more traditional and simplistic approaches such as logistic regression.

To address this, recent approaches have used Natural Language Processing (NLP), a field within machine learning that concentrates on text data with the goal of developing models that can understand human writing. Using NLP, researchers have used models that can identify whether VTE is present or absent from clinical notes (see Supplemental Table 1) [16–19]. These approaches have utilized tools that are rules based or advanced text miners; however, a major limitation in most of these approaches is that they use tools that are considered black box in which the architecture of the tools is not available. Some of these tools were from a commercialized source, further limiting the ability to replicate the methods. While the architecture in the tool published by Verma et al. is available, the tool used in this study used a rules-based NLP tool, rather than newer available tools [19].

In this investigation we develop ClotCatcher, which is a novel deep learning technology that incorporates the use of BERT (Bidirectional Encoder Representations from Transformers). BERT is a state-of-the-art model that incorporates several key advancements in the field of NLP that can be applied to textual data. First, BERT uses a transformer, which is a neural network used predominantly in NLP that allows the tool to weight certain parts of the sentence to predict the relationships between the words in the sentence. Having an improved understanding of the sentence allows the encoder to better detect key words allowing the tool to interpret hidden representation of the text, resulting in increased accuracy when decoding. These advancements led to the development of BERT, which achieved state-of-the-art results in NLP tasks. As a result, BERT has been utilized as a pre-trained base for constructing models that cater to specific contexts, such as biomedical texts, scientific publications, clinical notes, and patient information [20–22].

Secondly, BERT is also the first to use bidirectional models, in comparison to earlier models that trained unidirectionally, meaning that the tool only trained on language going in one direction (i.e., left-to-right). Bidirectional models, such as BERT, utilize a novel methodology by allowing input data to be input from either direction (i.e., both left-to-right and right-to-left), improving contextual understanding. Finally, BERT uses Masked Language Modeling (MLM), which is an approach to further improve prediction and contextual understanding by randomly masking words in the text and forcing the model to predict the word that is randomly masked. This allows the NLP tool to improve the contextual understanding of the text by looking at the surrounding sentence [23].

The objectives of our study were (1) to develop a deep learning tool using a deep learning model to detect VTE from clinical reports, (2) create a tool using open source methodologies that would not rely on black box algorithms, allowing for reproducibility, and (3) create a generalizable NLP tool by training the tool on datasets from two different hospital systems.

## Methods

### Study design

This study used a retrospective, observational design to create and validate ClotCatcher, a novel deep learning model to accurately adjudicate the presence or absence of venous thromboembolism (VTE) from radiology notes. This study was approved by the Emory University Institutional Review Board (STUDY00000302) under waiver of consent due to the retrospective nature of the study. This investigation was carried out in accordance to the Emory University Institutional Review Board guidelines and regulations.

### Data source

We included all radiology studies from patients who were admitted to either Emory University Hospital (EUH) (years 2014 – 2021) or Grady Memorial Hospital (GMH) (years 2014 – 2022). During the years noted, EUH used PowerChart™ from Cerner™ and GMH used Epic™. We selected only radiology studies that are used to evaluate for VTE (see Table 1). We did not include ventilation-perfusion scintigraphy (i.e. V:Q scans) as the results are given as a probability. We then randomly selected 5% of all studies from GMH and 2.5% of studies from EUH. The reports of all radiological studies were extracted into a document and a physician (JW) adjudicated whether VTE was present or absent. Notes which were not finalized or were incomplete were excluded from the study (*n* = 5).

**Table 1:** List of imaging studies selected

Imaging Studies Selected
Ultrasound – Bilateral Upper Extremity
Ultrasound – Right Lower Extremity
Ultrasound – Left Lower Extremity
Ultrasound – Bilateral Lower Extremity
Ultrasound – Right Lower Extremity
Ultrasound – Left Lower Extremity
Computed Tomography Chest w/ Intravenous Contrast (Pulmonary angiography)

### Cleaning and creating training dataset

The radiology reports were first extracted, and all text converted to lowercase to standardize the text. The reports from both GMH and EUH were then randomly split 80% to 20% (training to validation) for both hospitals. Randomly splitting the dataset into training and validation helps the model avoid overfitting, where the model performs well on the training set but poorly on new, unseen data. To maximize the potential from the training dataset, we performed data augmentation using paraphrasing, a technique that generates new versions of the text using different words and/or syntax, while still preserving the meaning of the original text. Two distinct strategies were developed, one with paraphrasing and one without paraphrasing. Paraphrasing can be done manually, however in our dataset, the Google PEGASUS model, a transformer-based neural network-based NLP tool was used which automates data paraphrasing. After applying the Google PEGASUS model to the training dataset, we generated 20 additional studies for each unique study, resulting in 21 × amplification of the training dataset.

**Fig. 1:**
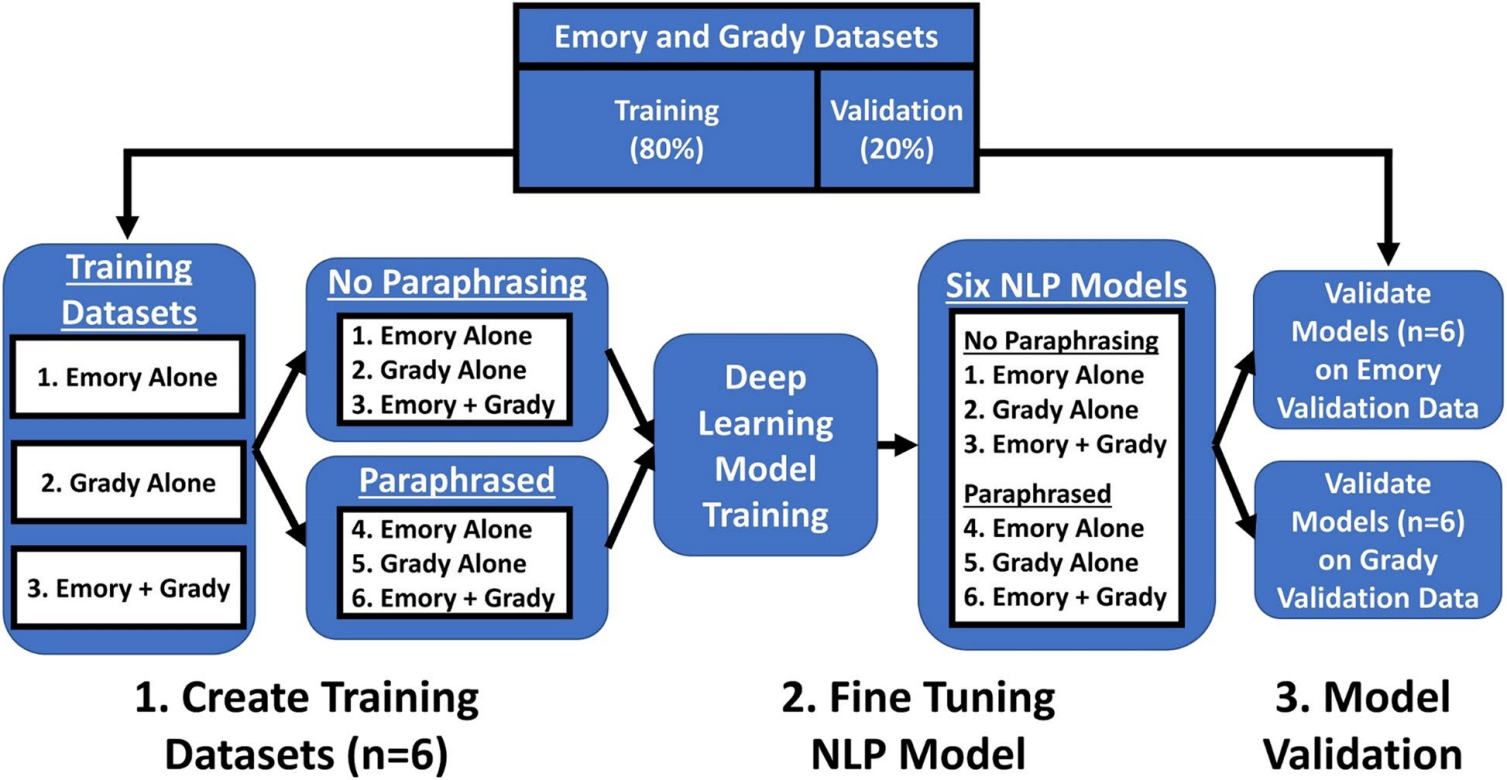
Overview of the architecture of the natural language processing pipeline

While we started with datasets from EUH and GMH, we created a third dataset by combining EUH and GMH resulting in three unique combinations – EUH alone, GMH alone, and EUH combined with GMH (see Fig. 1 for training dataset creation and model validation pipeline). We created six total training datasets, the initial three used the datasets without applying paraphrasing. The additional three were created after using data augmentation by applying paraphrasing, resulting in datasets that were 21 × the size of the original datasets [24].

### Clotcatcher tool development

The training datasets were then used to create the ClotCatcher tool by fine-tuning the BERT model to better adjudicate for the presence or absence of VTE based on the text available within the radiology report. The physician adjudicated result was considered the gold standard. Comparing the paraphrased dataset to the datasets not utilizing paraphrasing allowed us to evaluate the impact of data augmentation.

To evaluate the generalizability of ClotCatcher, we trained six deep learning models from six datasets (see Fig. 1). The first three were 1) EUH alone (without paraphrasing), 2) GMH alone (without paraphrasing), and 3) EUH combined with GMH (without paraphrasing). The final three datasets were the same as above, however with paraphrasing applied for data augmentation.

These six training datasets were then used to fine-tune the ClotCatcher tool to create six different deep learning models which were then validated on the EUH dataset alone and the GMH dataset alone. Key metrics to measure model performance such as sensitivity, specificity, F1 score, accuracy, and area under the receiver operating curve (AUC) were determined for each model and validation cohort combination. The architecture of the ClotCatcher tool can be seen in Fig. 1. All program codes were written for Python programming language (Version 3.9).

All ICD-9 and ICD-10 codes were extracted from each hospitalization for which the imaging report originated. ICD codes consistent with VTE were further determined based on previous literature [25]. Hospitalizations with ICD codes consistent with VTE were adjudicated as VTE positive by ICD Code. This was compared against physician adjudication, which is considered the gold standard. Key metrics to measure model performance such as sensitivity, specificity, F1 score, accuracy, and area under the receiver operating curve (AUC) were determined for GMH and EUH.

## Results

### Analytic cohort

**Fig. 2:**
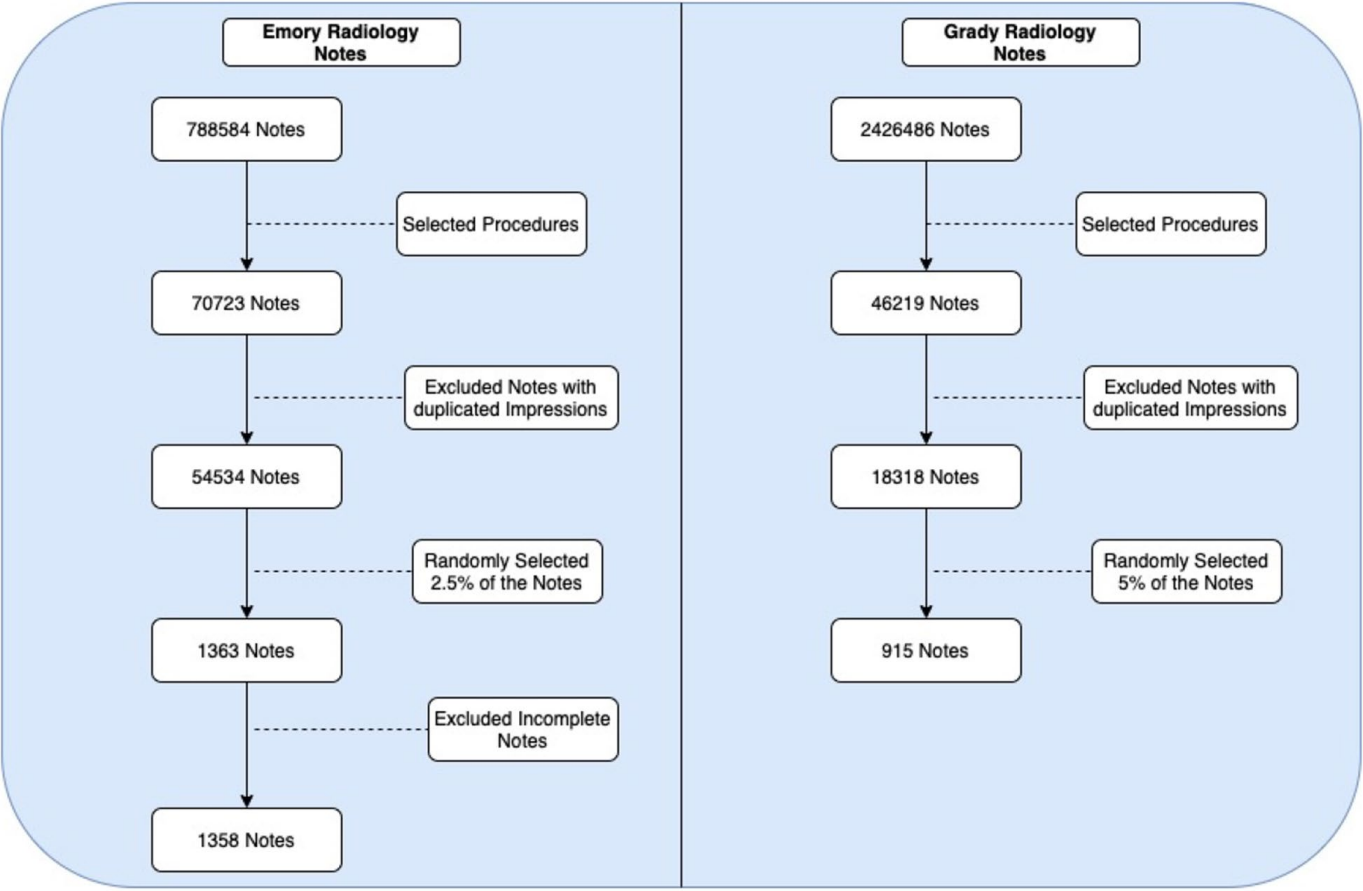
CONSORT diagram describing analytic cohort for studies

After applying appropriate inclusion criteria for radiological studies, we obtained 1358 studies from EUH and 915 studies from GMH (see Fig. 2). Both EUH and GMH datasets were randomly split into derivation (EUH *n* = 1086, GMH *n* = 732) and validation (EUH *n* = 272, GMH *n* = 183). The baseline characteristics for the patients stratified by hospital are listed in Table 2. Patients from GMH were younger (53.5 ± 16.9 vs 57.8 ± 17.5 years), less likely to be female (381/895 [32.5%] vs 754/1342 [56.2%]), and more likely to self-identify as African American (718 [80%] vs 613 [45.7%]). The proportion of ultrasound studies adjudicated to have VTE by a physician was 177/653 (27.1%) at EUH and 351/853 (41.1%) at GMH. The proportion of CT studies adjudicated to have VTE by a physician was 88/705 (12.5%) at EUH and 3/62 (4.8%) at GMH (Table 3). The median time to study ordered was 33.7 [Interquartile Range (IQR): 14.4, 139.2] hours, and 9.8 [IQR: 4.0, 93.6] hours for GMH (Table 2). When stratified by whether the study was positive for VTE (1) or not (2), the distribution plots are presented in Supplemental Fig. 3.

**Fig. 3:**
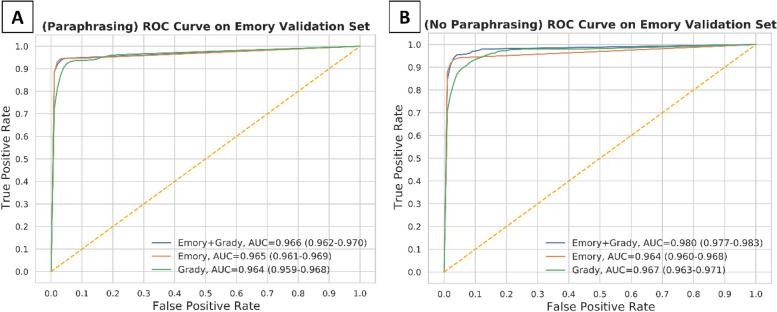
Model validation on the Emory dataset after training on either 1) Emory and Grady, 2) Emory alone, or 3) Grady alone dataset. This was evaluated with (3A) paraphrasing and (3B) without

**Table 2:** Baseline characteristics for analytic cohort by hospital

	**Emory University Hospital** (*n* = 1342)	**Grady Memorial Hospital** (*n* = 895)
**Age**	57.8 ± 17.5	53.5 ± 16.9
**Female,** n (%)	754 (56.2%)	381 (32.5%)
**Race**		
Caucasian	643 (47.9%)	83 (9.3%)
African American	613 (45.7%)	718 (80%)
Asian	35 (2.6%)	8 (0.9%)
**History of Diabetes**	427 (31.8%)	112 (12.5%)
**History of MI**	183 (13.6%)	29 (3.2%)
**History of CHF**	407 (30.3%)	100 (11.1%)
**History of PVD**	205 (15.3%)	21 (2.3%)
**Chronic Pulmonary Disease**	448 (33.4%)	117 (13%)
**CKD or Dialysis**	340 (25.3%)	96 (10%)
**History of Cancer**	246 (18.3%)	20 (2.2%)
**HIV/AIDS**	25 (1.9%)	17 (1.9%)
**Time from Admission to Radiological Study (hours)**	33.7 [14.4 – 39.2]	9.8 [4.0 – 93.6]

**Table 3:** Radiological study count and proportion positive for venous thromboembolism

	Emory (*n* = 1358)	Grady (*n* = 915)	Total (*n* = 2273)
**Ultrasound** **- Proportion Positive for VTE**	653 177 (27.1%)	853 351 (41.1%)	1506 528 (35.1%)
**CT Studies** **- Proportion Positive for VTE**	705 88 (12.5%)	62 3 (4.8%)	767 91 (11.9%)

### ClotCatcher tool validation

**Fig. 4:**
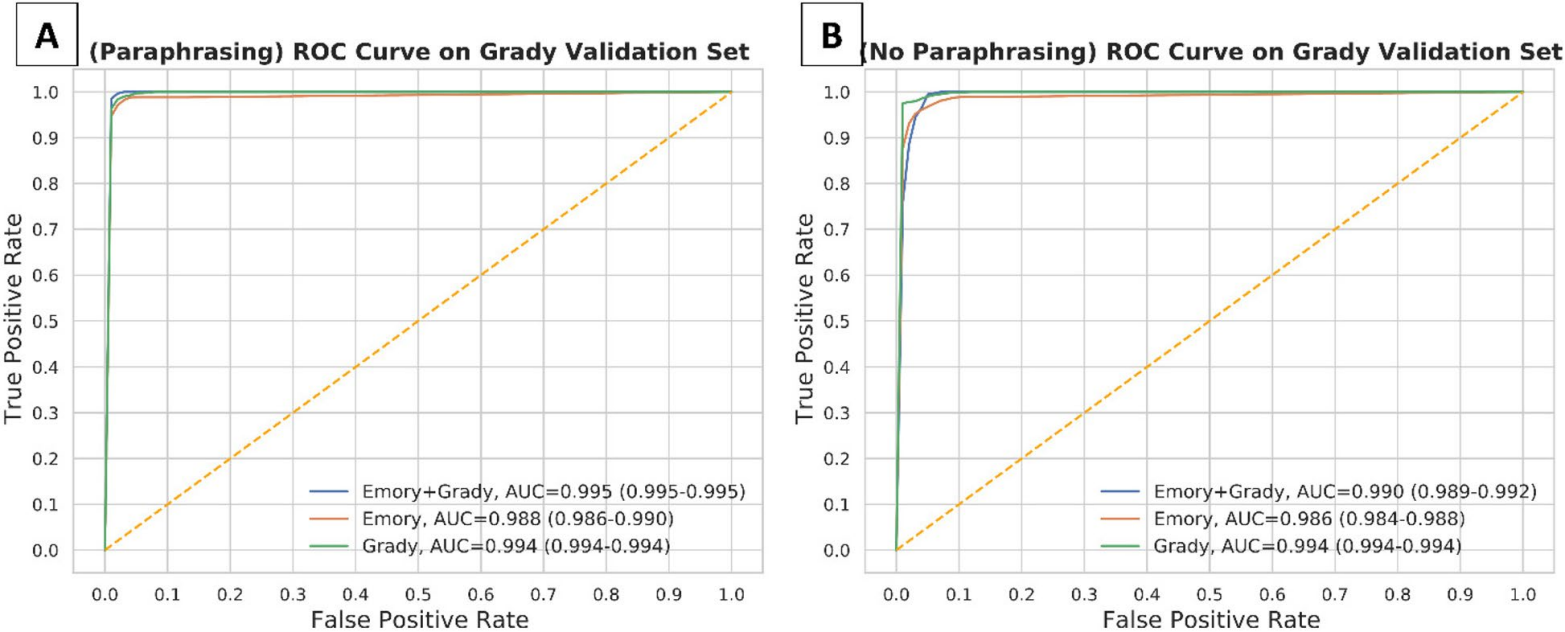
Model validation on the Grady dataset after training on either 1) Emory and Grady, 2) Emory alone, or 3) Grady alone dataset. This was evaluated with (4A) paraphrasing and (4B) without

We performed validation of all six deep learning models generated from ClotCatcher on the validation datasets from EUH and GMH separately. When validated on the EUH dataset, all six models produced excellent AUCs, ranging from 0.966 to 0.980 (Fig. 3, Table 4). Interestingly, the model with the highest AUC validated on the EUH dataset was fine-tuned on EUH combined with GMH data without paraphrasing (AUC 0.980, 95% Confidence Interval [CI]: 0.977 – 0.983). When validated on the GMH dataset, all six models also had excellent AUCs ranging from 0.988 – 0.995 (Fig. 4, Table 5). The model with the highest AUC validated on the GMH dataset was fine-tuned on EUH combined with GMH data utilizing paraphrasing for data augmentation. ClotCatcher performed better than using ICD to adjudicate whether VTE was present or not during the hospitalization across all metrics (Supplemental Table 2).

**Table 4:**
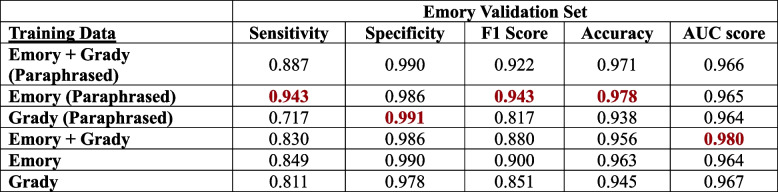
The sensitivity, specificity, F1 Score, accuracy and the area under the receiver operation curve (AUC) for the model validated on the Emory dataset with and without paraphrasing

Best in class metrics are bolded in red

**Table 5:**
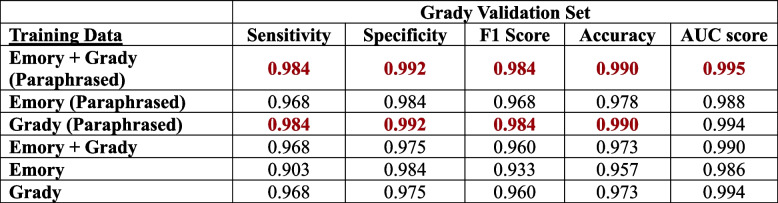
The sensitivity, specificity, F1 Score, accuracy and the area under the receiver operation curve (AUC) for the model validated on the Grady dataset with and without paraphrasing

Best in class metrics are bolded in red

## Discussion

In this study, we created ClotCatcher, a deep learning tool that was able to accurately and rapidly adjudicate the presence or absence of VTE from text in radiological reports. We obtained excellent results with our best models demonstrating an AUC of 0.980 on the EUH dataset and an AUC of 0.995 on the GMH dataset. Our approach is novel as it is the first study to use either BERT – which is considered state-of-the-art technology in natural language processing or the paraphrasing technique in developing a tool to detect VTE from radiology studies. Furthermore, the architecture of the ClotCatcher tool uses non-proprietary tools that are readily available. Previous approaches have relied on black box or commercialized tools that are not easily replicated. By combining these techniques into our pipeline, we have created a tool which is both powerful and replicable.

The high metrics of the ClotCatcher tool is in large part due to its use of BERT, which has been established as the industry standard in NLP and has found widespread use from medical research to Google searches. In our analysis, we used BioBert, the form of BERT pre-trained on biological data, which is the first use of BERT in adjudicating VTE from radiological studies. The significance of these tools are readily apparent when considering that only 2.5% of the Emory dataset and 5% of the Grady dataset was clinician adjudicated. Using ClotCatcher, we can now accurately and rapidly adjudicate all available studies within the EUH and GMH database.

There were important differences in the GMH and EUH patient populations (Table 2). These differences can be explained by the hospital locations and the communities which they serve. Patients at GMH were younger, more likely to be male, and had a higher proportion self-identify as African American. As patients are younger at GMH, younger patients generally have less comorbidities resulting in a lower prevalence of ICD codes in this population. Secondly, there are differences in VTE evaluation, at EUH, 48% (653/1358) of studies ordered were ultrasound, whereas at GMH, this was 93% (853/915). Secondly, while there are no readily apparent clinical differences to explain differences in ordering pattern, it is possible that differences in physician ordering practice could be driving these differences. In a retrospective observational study of emergency room physicians ordering CT studies to evaluate for VTE, the number of studies ordered by each physician varied greatly from 25 to 141 CT studies per physician [26].

Thirdly, the CT studies at GMH have a low positivity rate (4.8%) compared to EUH (12.5%). This can be in part explained that GMH is the only level one trauma center in metropolitan Atlanta. During the initial evaluation of complex poly-trauma, CT studies are often obtained to evaluate multiple pathologies simultaneously. Therefore, the pre-test probability for VTE in these situations are low, which can partially explain the low positivity rate. This seemingly low positive rate is consistent with existing literature demonstrating that among emergency room physicians ordering CT studies to evaluate for VTE, the proportion positive was 6.9% [26].

The tool directly addresses the inaccuracies from misclassification bias due to using ICD codes in studies investigating VTE. ICD codes are known to be inaccurate and previous literature demonstrated that the positive predictive value of a VTE ICD code to predict the presence of VTE diagnosed during hospitalization was poor, around 50%. By applying tools such as ClotCatcher to radiological studies, investigators will be able to accurately adjudicate the presence of VTE use radiological studies. This will allow for more accurate epidemiological studies and reduce misclassification bias.

In addition to accurate adjudication, all radiological studies have an associated time stamp which will provide the time VTE was diagnosed on a radiological study. When relying on traditional methods which use ICD codes, lack of time data limited analyses to more simplistic methods such as logistic regression. Incorporating the time of the event will allow for more sophisticated analyses such as Cox-proportional hazard modeling and Kaplan Meier analysis. Furthermore, these modeling approaches will form the basis to create automated individualized risk prediction scores for patients admitted to the hospital. This is particularly relevant for patients who have their chemoprophylaxis for VTE held as applying individualized risk prediction models can alert clinicians to consider starting chemoprophylaxis in patients with a high risk for in-hospital VTE.

An important aspect of applying machine learning models to healthcare data is the concept of generalization, which refers to the ability of a model to accurately predict outcomes from data that is different from the source population it was originally trained on. In healthcare, data limitations often constrain researchers to use data from the same hospital for training and testing; however, given the increasing popularity of machine learning models in healthcare, it is crucial to investigate the performance of models on data sourced from different hospitals and healthcare systems [27, 28]. Our model had excellent performance on both the EUH and the GMH datasets, demonstrating that our model is not limited by the origin of the training data. Furthermore, the two hospitals used different electronic medical records during the study period which further supports the generalizability of our model. The slight variation in the metrics could be due to different documentation standards across hospitals. Future directions would include replicating our analysis using additional hospital systems.

There were several limitations. We used a subset of the available data, given limitations in time and resources in utilizing physician adjudication of the radiological studies. We were also limited to studies that were commonly used to identify VTE, thereby missing incidental VTE found by other studies (i.e., portal vein thrombus, splanchnic vein thrombus, etc.). We also excluded ventilation-perfusion scans from this study given that the interpretation of these studies is usually provided as probabilities. Finally, radiologists at GMH can have appointments at EUH as well and therefore there may be similarities between these two hospitals in producing radiological reports.

In conclusion, we present the results from ClotCatcher, a novel deep learning tool to accurately and rapidly adjudicate the presence or absence of VTE from radiological reports. The tool can be readily replicated using existing open-source tools such as BERT and the paraphrasing technique. Validation of this ClotCatcher serves as the foundation for improving identification of VTE cases from large databases.

### Supplementary Information

**Additional file 1: Supplemental Table 1.** Summary of published natural language processing models to adjudicate the presence or absence of deep vein thrombosis from radiological studies.** Supplemental Figure 1.** Model calibration on the Emory dataset after training on either 1) Emory and Grady, 2) Emory alone, or 3) Grady alone dataset. This was evaluated with (1A) paraphrasing and (1B) without.** Supplemental Figure 2.** Model calibration on the Grady dataset after training on either 1) Emory and Grady, 2) Emory alone, or 3) Grady alone dataset. This was evaluated with (1A) paraphrasing and (1B) without.** Supplemental Figure 3.** Distribution plots for Time from Admission to Radiological Study Order.** Supplemental Table 2.** Metrics for VTE positive using ICD Codes.

## Data Availability

Data is available upon reasonable requests. For original data, please contact rkamaleswaran@emory.edu.
