# Supplementary material for: ClotCatcher: a novel natural language model to accurately adjudicate venous thromboembolism from radiology reports
Source: BMC Med Inform Decis Mak. 2023 Nov 16;23:262. doi: 10.1186/s12911-023-02369-z (PMC10652606; doi:10.1186/s12911-023-02369-z)
Supplement: Supplementary file 1 — Additional file 1: Supplemental Table 1. Summary of published natural language processing models to adjudicate the presence or absence of deep vein thrombosis from radiological studies. Supplemental Figure 1. Model calibration on the Emory dataset after training on either 1) Emory and Grady, 2) Emory alone, or 3) Grady alone dataset. This was evaluated with (1A) paraphrasing and (1B) without. Supplemental Figure 2. Model calibration on the Grady dataset after training on either 1) Emory and Grady, 2) Emory alone, or 3) Grady alone dataset. This was evaluated with (1A) paraphrasing and (1B) without. Supplemental Figure 3. Distribution plots for Time from Admission to Radiological Study Order. Supplemental Table 2. Metrics for VTE positive using ICD Codes. [file 12911_2023_2369_MOESM1_ESM.docx]

**ClotCatcher: A Novel Natural Language Model to Accurately Adjudicate Venous Thromboembolism from Radiology Reports**

**Supplemental Data**

Supplemental Table 1: Literature review on NLP in identifying VTE

Supplemental Figure 1: Calibration plots for model validated on Emory dataset

Supplemental Figure 2: Calibration plots for model validated on Grady dataset

Supplemental Figure 3: Distribution plots for Time from Admission to Radiological Study Order

Supplemental Table 2: Metrics for VTE positive using ICD Codes

Supplemental Table 1: Summary of published natural language processing models to adjudicate the presence or absence of deep vein thrombosis from radiological studies.

|  | **Data Source** | **Training Sample** | **Reference Standard** | **Validation Sample** | **Method** |
| --- | --- | --- | --- | --- | --- |
| **Galvez et al**  **(2017)** | BLE Ultrasound | None | Physician Adjudicated | 250 | Reveal NLP: inference rule based tool |
| **Woller et al (2021)** | All Ultrasound  CTA-PE Studies  VQ Scans | None | Physician Adjudicated | 300 | NLP “text mining” tool. |
| **Shi et al**  **(2021)** | Clinical Notes  All Ultrasound  CTA-PE Studies | 6735 | Coder/Biller Adjudicated | 19,209 | EasyCIE-PEDVT: A rule based NLP Tool |
| **Verma et al**  **(2022)** | Ultrasound  CTA-PE Studies  VQ Scans | 1986 | Physician Adjudicated | 1,183 | “simpleNLP Tool”  Rule based NLP Tool |

Supplemental Figure 1: Model calibration on the Emory dataset after training on either 1) Emory and Grady, 2) Emory alone, or 3) Grady alone dataset. This was evaluated with (1A) paraphrasing and (1B) without.


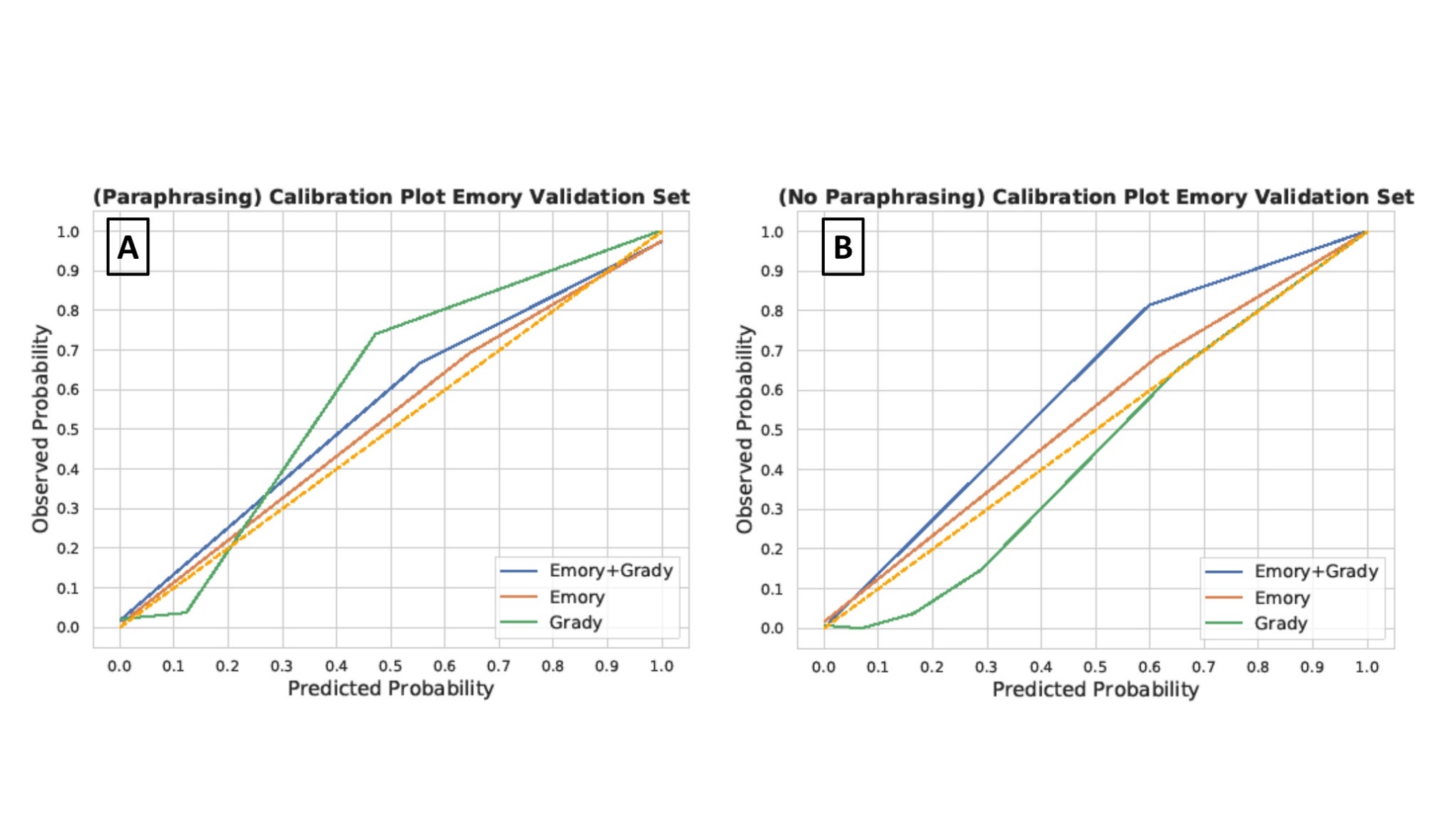


Supplemental Figure 2: Model calibration on the Grady dataset after training on either 1) Emory and Grady, 2) Emory alone, or 3) Grady alone dataset. This was evaluated with (1A) paraphrasing and (1B) without.


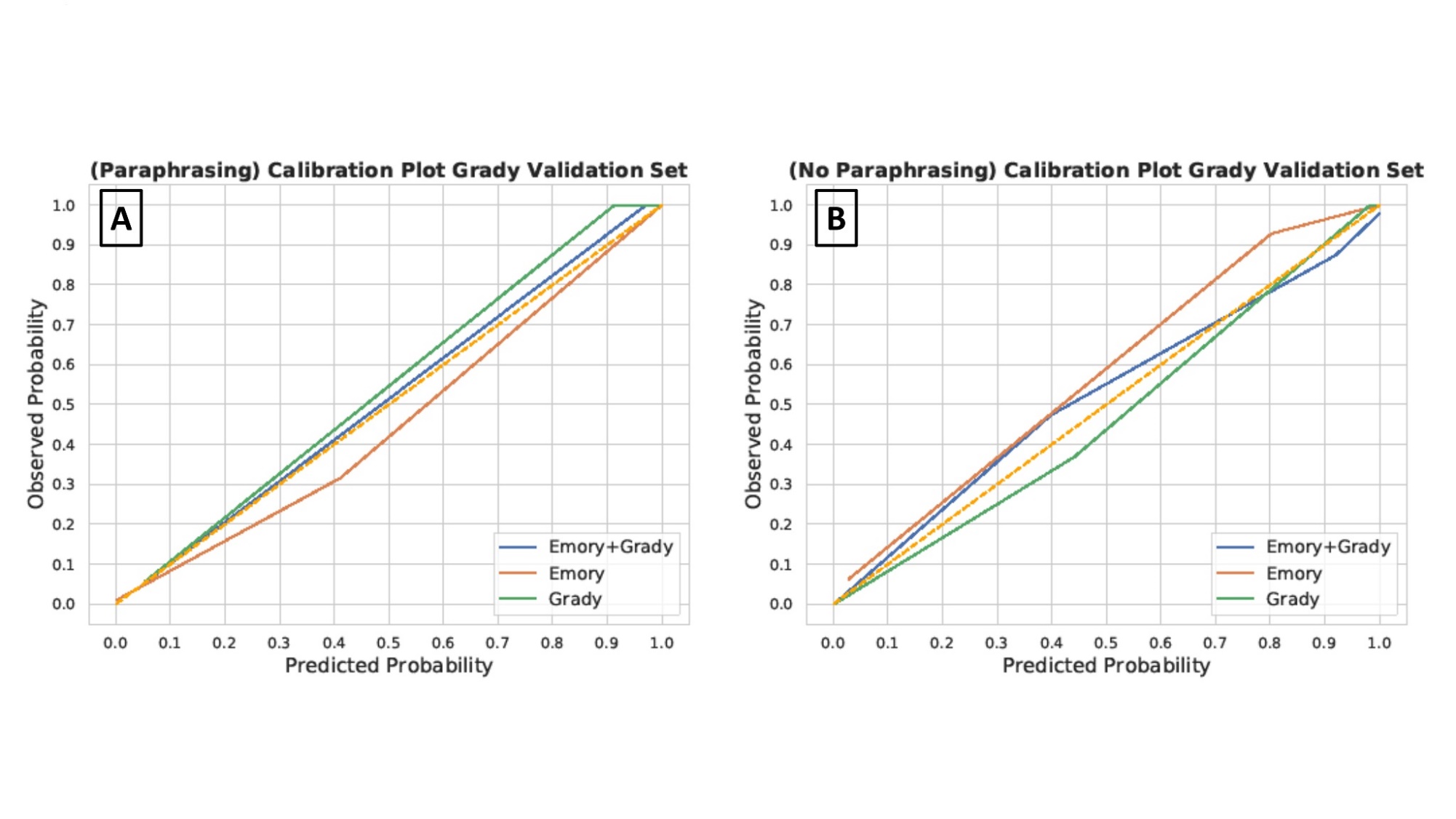


Supplemental Figure 3: Distribution plots for Time from Admission to Radiological Study Order


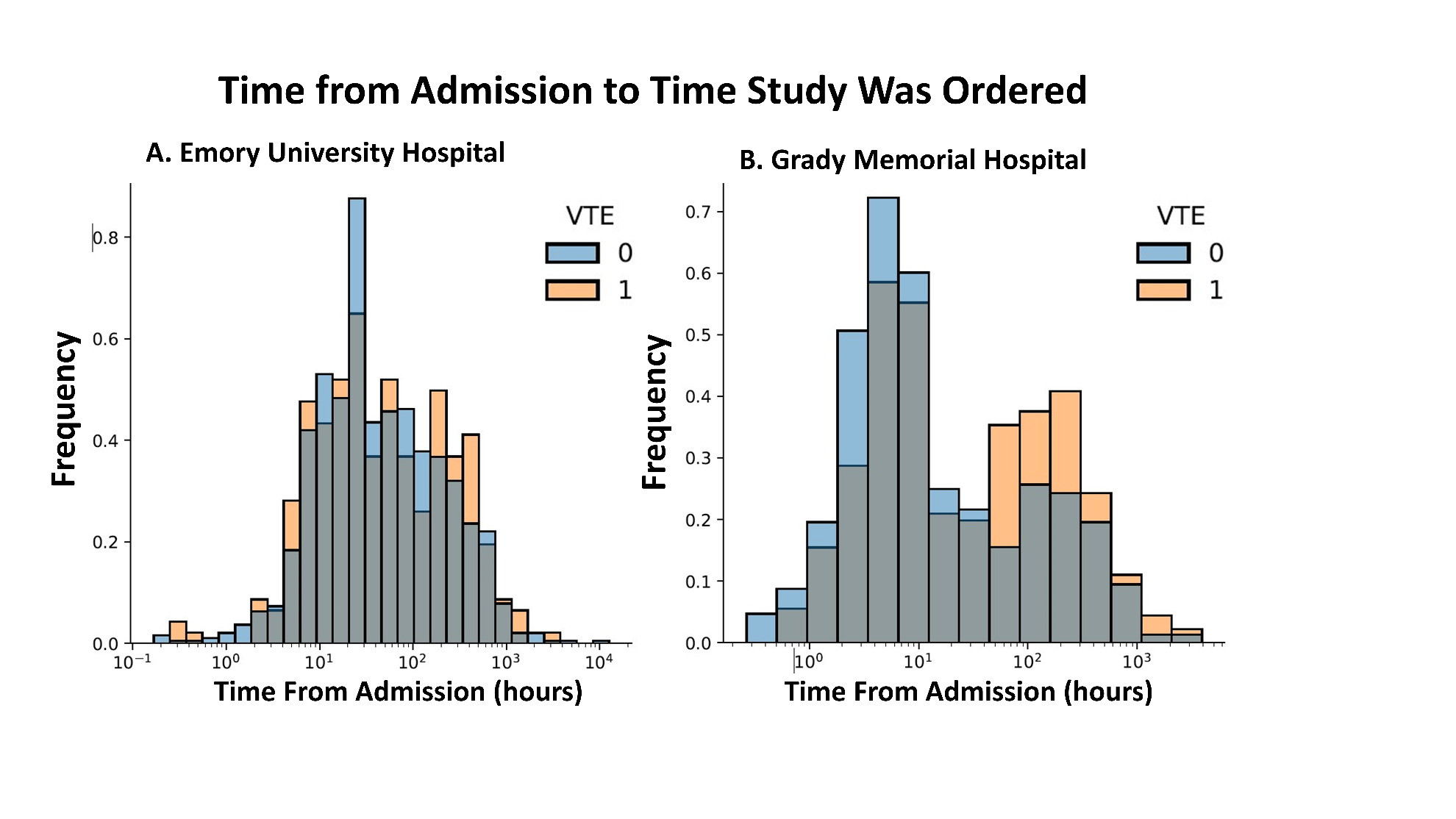


Supplemental Table 2: Metrics for VTE positive using ICD Codes

|  | **Sensitivity** | **Specificity** | **F1 Score** | **Accuracy** | **AUC Score** |
| --- | --- | --- | --- | --- | --- |
| **EUH** | 0.89 | 0.80 | 0.65 | 0.82 | 0.84 |
| **GMH** | 0.22 | 0.98 | 0.34 | 0.75 | 0.60 |

Abbreviation: EUH: Emory University Hospital, GMH: Grady Memorial Hospital
